# Rice with Multilayer Aleurone: A Larger Sink for Multiple Micronutrients

**DOI:** 10.1186/s12284-021-00543-3

**Published:** 2021-12-13

**Authors:** Ronald Yu, Xiaoba Wu, Jinxin Liu, Crispin A. Howitt, Anthony R. Bird, Chun-Ming Liu, Philip J. Larkin

**Affiliations:** 1 CSIRO Agriculture, GPO Box 1600, CSIRO, Building 801, CSIRO Black Mountain, Clunies Ross Street, Canberra, ACT 2601 Australia; 2 CSIRO Health and Biosecurity, PO Box 10041, Adelaide, SA 5000 Australia; 3 Key Laboratory of Plant Molecular Physiology, Institute of Botany, Chinese Academy of Sciences, Beijing, China

**Keywords:** Aleurone bran, Fibre, Minerals, Multiple micronutrients, Nutrients, Phytate

## Abstract

Diet-related noncommunicable diseases impose a heavy burden on human health worldwide. Rice is a good target for diet-related disease prevention strategies because it is widely consumed. Liu et al*.* (Proc Natl Acad Sci USA 115(44):11327–11332, 2018. https://doi.org/10.1073/pnas.1806304115) demonstrated that increasing the number of cell layers and thickness of putative aleurone in *ta2-1* (*thick aleurone 2-1*) mutant rice enhances simultaneously the content of multiple micronutrients. However, the increases of aleurone-associated nutrients were not proportional to the increases in the aleurone thickness. In this study, first, cytohistological analyses and transmission electron microscopy demonstrated that the multilayer in *ta2-1* exhibited aleurone cell structural features. Second, we detected an increase in insoluble fibre and insoluble bound-phenolic compounds, a shift in aleurone-specific neutral non-starch polysaccharide profile, enhancement of phytate and minerals such as iron, zinc, potassium, magnesium, sulphur, and manganese, enrichment of triacylglycerol and phosphatidylcholine but slight reduction in free fatty acid, and an increase in oleic fatty acid composition. These findings support our hypothesis that the expanded aleurone-like layers in *ta2-1* maintained some of the distinctive aleurone features and composition. We provide perspectives to achieve even greater filling of this expanded micronutrient sink to provide a means for multiple micronutrient enhancements in rice.

**Supplementary Information:**

The online version contains supplementary material available at 10.1186/s12284-021-00543-3.

## Background

### What We Know?

Globally, diet-related noncommunicable diseases (NCDs) have significant negative impacts on human health and create an ever-growing burden on health systems. According to the World Health Organization in 2014, chronic diseases such as cardiovascular diseases, cancers, and type II diabetes caused more than 63% of total deaths worldwide (Armstrong et al. 2014; GBD Risk Factors Collaborators 2016). Recognizing the compelling threat posed by NCDs, diet-related prevention strategies have become a top priority for all stakeholders with evident social and economic advantages compared to efforts to cure these conditions (Lachat et al. 2013; World Health Organization 2005). The two central concepts behind the work presented here are that: (1) health outcomes can be achieved more readily by improving the composition of staple food; and (2) it is possible to co-ordinately improve multiple nutritional factors in order to achieve desired outcomes.

Rice represents an ideal target for such compositional intervention, being a widely consumed staple in both developing and developed countries. Among the top five most populated countries, China, India, and Indonesia are the developing countries that rely heavily on rice as a staple food (FAOSTAT 2019). White rice is the most commonly consumed type and a good source of dietary energy but poor in many essential nutrients including dietary fibre, antioxidants, phenolic compounds, minerals, and vitamins. Improving the content of any one of these alone would likely be insufficient to reduce the risk of any specific type of chronic disease (Borresen and Ryan 2014; Henderson et al. 2012; Kahlon 2009; Nagendra Prasad et al. 2011). On the other hand, national campaigns have been initiated to promote brown rice (wholegrain rice) consumption. In the Philippines, a campaign called “BeRICEponsible” was initiated in 2014 to promote the health benefits associated with brown rice and to encourage brown rice consumption (Mohan et al. 2017). In comparison with white rice, brown rice is a good source of many essential nutrients because of the presence of aleurone in bran (mixed tissues of pericarp, testa, aleurone, and embryo).

In rice, the bran tissues provide about 40% of the total minerals, 90% of the total phosphorus, 50% of the total niacin (Vitamin B3), 50% of total lipid, and about 50% of the total phenolic compounds of the whole rice caryopsis (Goufo and Trindade 2014; Kik and Williams 1945; Resurreccion et al. 1979). Along with the embryo, aleurone is the major contributor to the micronutrients of bran, and the aleurone represents more than 66% of rice bran fraction (Taira 1995). Aleurone is the outermost cell layer of rice endosperm and very distinctive from the inner starchy endosperm (Becraft 2001; Wu et al. 2016). In rice, the number of aleurone layers can influence the quantities of important micronutrients such as iron and zinc (Sellappan et al. 2009; Yu et al. 2014). In our previous work we isolated and characterised in molecular genetic detail a rice mutant *thick aleurone 2-1* (*ta2-1*) with thicker aleurone phenotype; a point mutation in the 14th intron of the DNA demethylase *OsROS1* led to alternative splicing responsible for the new phenotype (Liu et al. 2018). In comparison with single-cell layer aleurone in the wild type, *ta2-1* has an average of 4.8 aleurone cell layers and up to 10 cell layers in some regions of the grain. Increases in nutritional components such as protein, lipid, starch, minerals, dietary fibre, antioxidants, and different vitamins were detected (Liu et al. 2018). However, the increases for most of the aleurone-associated nutrients and phytochemicals were not proportional to the increases in aleurone thickness. This was particularly evident for the important minerals of iron and zinc. This may suggest that the observed extra aleurone cells may not express the full biosynthetic functionality of aleurone cells, and/or the full allocation functionality of components such as iron and zinc. In the case of minerals, the uptake, transport and/or loading capacity may affect their final contents in aleurone.

### What We Don’t Know?

In this study, our aim is to test whether the multilayer aleurone in *ta2-1* maintains the various aleurone-like identities, and acts as a source for multiple nutritional enhancements. Two approaches were developed for this aim. First, different microscopic techniques were used to visualize the spatial and temporal distribution of different cellular and subcellular elements of aleurone cells in *ta2-1* during caryopsis development. Second, additional nutritional and compositional analyses were conducted to assess whether different nutritional elements such as dietary fibre and phenolic compounds, fatty acids, minerals, and phytate in the multi-layer aleurone of *ta2-1* follow the same aleurone-specific distribution patterns as in the wild-type. The interaction of minerals and phytate and their potential effect on human nutrition will be discussed. The aim of this study is to further improve the biofortification strategy in the *ta2-1* rice background and pave the way for similar improvements in other cereals.

## Materials and Methods

### Statistical Methods and Assumptions

Tukey’s Honestly Significant Difference (Tukey’s HSD) test was used to test the hypothesis that the nutrient content of *ta2-1* mutant rice was different from wild-type with the null hypothesis as no difference (Kirk 1968; Tukey 1953). Statistical analyses were conducted using R and all the results reported as significant were those with significance level of *P* < 0.05 (R Core Team 2013). The exact p-value of all the statistical analyses in this study can be found in the file of Figs. 5, 6, 7, 8, 9, 10, 11 and 12_RawData.xlsx in the supplementary information.

### Cultivation of the Wild-Type and *ta2-1* Mutant Rice for Analysis

Rice mutant *ta2-1* was backcrossed three times (BC3) to remove unwanted ethyl methanesulfonate mutations, following by two rounds of self-pollination and selection to identify lines homozygous and stable for the thick-aleurone phenotype. The rice grains of BC3F3 homozygous *ta2-1* and wild-type Zhonghua 11 (ZH11) were sown side by side in the field for the grain increase in Haidian, Beijing, Peoples Republic of China (39.95°N, 116.38°E). In this study, 50 individual plants from *ta2-1* and wild-type ZH11 were harvested and analysed. The hull (palea and lemma) of the rice caryopsis was removed. No polishing was done so that the bran layer (including the rice aleurone layers) was maintained. Rice caryopses from 50 individual plants from *ta2-1* and wild-type were milled to wholegrain rice flour by Laboratory Mill 3100 (LM3100, Perten) in the same day and under similar temperature and humidity. This milled wholegrain rice flour sample was used for all the nutritional and biochemical analyses. This treatment has advantage that it can mimic the industrial milling procedure which requires a relatively large amount of rice caryopses as starting materials. However, this treatment also results in a statistical limitation of low biological replicate. All the replicates used in my study represent technical replicates from one biological replicate of a single field trial. BC3F4 rice seed of *ta2-1* and ZH11 lines has been analysed in Liu et al. (2018) and BC3F5 seed was used for the current study.

### Sudan Red Staining of Aleurone Tissue

Stain solution was prepared by dissolving 1 g of Sudan red IV in 50 ml of polyethylene glycol solution (202398, Sigma-Aldrich), incubated at 90 °C for one hour, and mixed with equal volume of 90% glycerol. After removing the fruit coat (palea and lemma) of each grain, mature rice grains were incubated in distilled water for five hours and then sectioned transversely or longitudinally using a razor blade. Sections were stained in Sudan red solution at room temperature for 24 to 72 h, followed by counterstaining with Lugol’s staining solution (32922, Sigma-Aldrich) at room temperature for 20 min and observed under stereomicroscope (M80, Leica Microsystems) (Sreenivasulu et al. 2010).

### Light Microscopic Observation Coupled with Calcofluor White or Periodic Acid-Schiff and Coomassie Brilliant Blue Staining

Rice grains were fixed in formalin-acetic acid-alcohol solution (60% ethanol, 5% glacial acetic acid and 2% formaldehyde), degassed for one hour, dehydrated in a series of alcohol solutions containing 70%, 80%, 95%, and 100% ethanol, infiltrated in LR white resin (14380, Electron Microscopy Sciences), and polymerized for 24 h at 60 °C. Microtome sectioning was done by Leica UC7 microtome (UC7, Leica Microsystems).

In Calcofluor white staining, sections were stained in 0.01% Calcofluor white solution (18909, Sigma-Aldrich) at room temperature for 2 min and examined by light microscopy.

In Periodic acid-Schiff (PAS) and Coomassie brilliant blue (CBB) staining, the fixed sections on slides were incubated in preheated 0.4% periodic acid (375810, Sigma-Aldrich) at 57 °C for 30 min, followed by rinsing in distilled water for three times. Schiff reagent (3952016, Sigma-Aldrich) was applied and the slides were incubated at room temperature for 15 min, following by rinsing three times in distilled water. The sections were then incubated in 1% Coomassie blue R-250 (20278, ThermoScientific) at room temperature for 2 min and rinsed three times with distilled water. Sections were dehydrated in a series of alcohol solutions of 30%, 50%, 60%, 75%, 85%, 95%, and 100% ethanol for 2 min each, followed by clearing of each slide in 50% xylene and 100% xylene solution (534056, Sigma-Aldrich) for 2 min each. The sections were then mounted on coverslips with Eukitt® quick hardening mounting medium (03989, Fluka) and observed under a light microscope.

### Transmission Electron Microscopic Observation

After removing the fruit coat (palea and lemma) of each grain, mature rice grains were transversely sectioned by a razor blade. Sectioned rice grain was fixed in 2.5% glutaraldehyde in a 0.1 M sodium phosphate buffer (pH 7.0) for one hour, washed in the same buffer, and incubated at room temperature for 30 min for three times. The fixed rice grain was then undergone post-fixation in 1% osmium tetraoxide at 4 °C for 16 h (overnight). After fixation, samples were washed in sodium phosphate buffer (pH 7.0) for 30 min for three times, followed by dehydration in a series of alcohol solutions of 30%, 50%, 60%, 75%, 85%, 95%, and 100% ethanol for 10 min each. The dehydrated samples were then infiltrated with acetone and Spurr® solution (14300, Electron Microscopy Sciences) in a series of solutions with acetone to Spurr ratio of 1:0, 2:1, 1:1, 1:2, and 0:1 for four hours each, and embedded at 60 °C for 16 to 24 h. Embedded samples were sectioned, stained, and observed under transmission electron microscope (1230, JEOL), as reported by Roland and Vian (1991).

### Aleurone Grain Measurement by Fiji ImageJ

TEM images were processed by the image processing package Fiji in ImageJ (Schindelin et al. 2012). In the Fiji package of ImageJ, the actual dimensions of TEM image were calibrated based on the scale bar, then threshold between 196 and 255 was selected to represent the aleurone grain area in wild type and *ta2-1*. Aleurone grain area was measured in terms of absolute area (µm^2^) or percentage area to the whole image (%).

### Total Mineral Content Estimation and Minerals Composition Measurement

The total mineral content of samples was measured by ash assay according to AOAC Method 923.03 (AOAC International 2016). In ash assay, about 2 g of desiccated rice flour was heated at 540 °C for 15 h and the mass of ash residue was then weighed.

Minerals composition was determined by ICP-OES (inductively coupled plasma optical emission spectrometry) (Zarcinas and Cartwright 1983; Zarcinas et al. 1987). In minerals composition assay, about 0.5 g of rice flour was digested using tube block digestion with 8 M nitric acid at 140 °C for eight hours. Zinc, iron, potassium, magnesium, phosphorus and sulphur content were then analysed using ICP-OES at CSIRO, Urrbrae, Adelaide, South Australia, at Waite Analytical Service (University of Adelaide, Waite, South Australia, Australia).

### Total Phytic Acid Content

Determination of the phytate content of the flour samples was conducted based on the method of Harland and Oberleas (1986), as described in AOAC Method 986.11 (AOAC International 2016; Harland and Oberleas 1986). Briefly, 0.5 g cereal flour sample was weighed, extracted with 2.4% hydrochloric acid for one hour, and centrifuged. The supernatant was diluted and subjected to anion exchange column (500 mg, 59822065, Agilent Technologies) to remove the non-phytate elements. Phytate bound to the column was then eluted with 2 M hydrochloric acid. Phosphorous levels were determined by spectrophotometer using the molybdate and sulphonic acid colouring method with absorbance readings at 640 nm. Phytate was calculated using the following formula:

$${\text{Phytate}}\,({\text{mg}}/{\text{g}}) = {\text{Pconc}}*{\text{V1}}*{\text{V2}}/({1}000*{\text{sample}}\,{\text{weight}}*0.{282})$$

where P concentration is the concentration of phosphorous (μg/ml), as determined by spectrophotometry, V1 is the volume of the final solution, V2 is the volume of the extracted phytate solution, and 0.282 is the phosphorus to phytate conversion factor.

### Fibre Content

Total dietary fibre was measured according to AOAC Method 985.29 and soluble and insoluble fibre according to AOAC Method 991.43 (AOAC International 2016).

Samples of 1 g of rice flour were undergone sequential enzymatic digestions of heat stable α-amylase (300 U/ml, E-BLAAM, Megazyme) and amyloglucosidase (3300 U/ml, E-AMGDF, Megazyme) to hydrolyse the starch content, followed by protease (350 tyrosine units/ml, E-BSPRT, Megazyme) to depolymerise the protein content. After the enzymatic digestions, different extraction and purification methods have applied to yield the total-, soluble- and insoluble-fibre fractions. Direct precipitation of sample after enzymatic digestions yielded the total-fibre; filtration of the enzymatic digested sample through Celite (61790-53-2, Sigma-Aldrich)-embedded fritted glass crucible, following by ethanol precipitation of the filtrate yielded the soluble-fibre while the residue after filtration represented the insoluble-fibre. After the enzymatic digestions and fibre extraction, the total-, soluble- and insoluble-dietary fibres were determined by the gravimetric measurements of the residue dry mass of the total-precipitated-residue, the filtrate-precipitated-residue and the residue after digestion respectively, with the correction from the parallel measurement of ash and protein content of the triplicate of each sample.

### Total Neutral Non-starch Polysaccharide

Neutral non-starch polysaccharide (NNSP) was measured by the gas chromatographic procedure according to AOAC Method 994.13, as detailed by Theander et al*.* with slight modification (AOAC International 2016; Theander et al. 1995). Briefly, the total-dietary fibres extracted from total dietary fibre assay were hydrolysed with sulfuric acid. The neutral sugars released were then reduced by potassium borohydride solution and acetylated by acetic acids to alditol acetates, which are then quantified by gas chromatography. Gas chromatography conditions: BPX70 column (30 m x 320 µm × 0.25 µm); injection 0.5 μl; inlet at 240 °C; 20:1 split; 1.62 ml/min constant flow; oven at 180 °C for 2 min, 12 °C/min to 230 °C and hold for 8 min; flame ionization detector at 250 °C; quantification against internal allose standard.

### Total Phenolic Compounds

Total phenols assay by FCR (Folin–Ciocalteu reagent) is an electron transfer-based assay, which estimates the amount of antioxidant based on the reducing power (willingness to donate electrons) of the antioxidants against oxidant probe molybdates, quantifies the reducing power with gallic acid standards, and presents as the value of gallic acid equivalents. Combined with the differential extractions of the free-, conjugated-, bound-, and total-fractions, FCR assay is good at determining the antioxidant capacity of different groups of phenolic compounds.

Total phenolic compounds content, as well as phenolic compounds in the free-, conjugated-, and bound-states were determined according to the method described by Li et al*.* with minor modifications (Li et al. 2008). Briefly, these four types of phenolic compounds were extracted from 100 mg samples with different extraction methods. Total-phenolic compounds were determined using 100 mg of samples, adding 200 µl 80% methanol, followed by alkaline hydrolysis with (2 M) sodium hydroxide; free-phenolic compounds were represented by the 80% methanol extraction of the 100 mg samples; conjugated-phenolic compounds were the alkaline hydrolysed products from the 80% methanol extract; bound-phenolic compounds were the alkaline hydrolysed products from the residues followed by the methanolic extraction of the free-phenolic compounds.

The amount of phenolic compounds in the treated/extracted samples was measured using FCR assay with reference to standard curve of known gallic acid concentrations. 1 ml of standards were added to 4 ml glass tubes. For test samples, 100 μl aliquots of thoroughly mixed samples were added to 900 μl water in 4 ml glass tubes. 100 ml of FCR (F9252, Sigma-Aldrich) was then added to each tube and vortexed immediately. 700 μl sodium bicarbonate solution (1 M) was added after 2 min and mixed by vertexing. Each solution was incubated at room temperature in the dark for one hour. Absorbance was read at 765 nm. Results were expressed in μg gallic acid equivalents/g sample.

### Folate Content

Total folate (Vitamin B9) was measured by commercially available VitaFast Folic acid kit (Folic Acid AOAC-RI, 100903, R-Biopharm) according to AOAC method 2004.05 as described by DeVries et al*.* (2005). The method incorporated the in vitro enzymes digestion and the growth response of the *Lactobacillus* to folate concentration in culture medium. First, pancreatin digestion was used for the release of the food matrix-bound folic acid. About 1 g of rice flour and 20 mg pancreatin were added and filled up to 40 ml with phosphate buffer (0.05 M, 0.1% ascorbate, pH7.2), and the sample was incubated at 37 °C for two hours in dark. These allowed the digestion of food matrix by protease and amylase in pancreatin and the hydrolyzation of polyglutamates to diglutamates by pancreatic conjugase to release food-bound folate which can be metabolized by *Lactobacillus*. After the incubation, the 1.5 ml reaction was centrifuged at 8000×*g* for 5 min, diluted to 10 ml with sterile distilled water, heated at 95 °C for 5 min, and filtered through a 0.2 µm filter to get rid of the insoluble or undigested suspensions in the sample. The filtrate was then diluted in several dilutions, added into the wells of a microtiter plate coated with *Lactobacillus casei* subspecies *rhamnosus*, and incubated at 37 °C for 44 to 48 h. After the incubation, the OD at 610–630 nm of the sample was measured and compared with the folate calibration standard. As the basal culture medium is lack of folate, the growth of the coated *Lactobacillus*, represented by the increase in turbidity of bacterial culture, is positively correlated with the extracted folate content in the sample. The folate concentration of the sample can be measured by comparing difference between the absorbance of the test sample with the calibration standards at OD 610–630 nm.

### Total Lipid Content

Total lipid was measured according to AOAC Method 983.23 (AOAC International 2016). Sample of 5 g rice flour was incubated with 1% Clarase (MC23.31, Southern Biological) in 0.5 M sodium acetate solution at 45 °C for one hour. Lipids were extracted from the sample into chloroform/methanol by multiple extractions. The samples were then subjected to homogenization with multiple additions of chloroform/methanol. After centrifugation of the mixture into separate phases, the chloroform/methanol fraction was removed and dried at 101 °C for 30 min to recover the lipid. The total lipid in the sample was represented by the mass of residue after drying.

### Triacylglycerol, Free Fatty Acid, and Phosphatidylcholine Fractionation and Quantification

According to the methodologies described by Liu et al*.*, the extraction of total lipid, the fractionation of neutral lipid of mainly triacylglycerol (TAG), free fatty acid (FFA), and polar lipid of mainly phosphatidylcholine (PC), and the quantification of lipid were carried out (Liu et al. 2017). Thin layer chromatography (TLC) was first carried out to separate the neutral lipid and free fatty acid in solvent matrix of hexane:diethyl ether:acetic acid in 70:30:1 volume ratio. Then, another TLC was conducted in solvent matrix of chloroform:methanol:acetic acid:distilled water in 90:15:10:3 to separate the polar lipid PC. After TLC, samples were collected and lipid content in the fraction was extracted. The lipid fractions were undergone fatty acid methyl esters (FAME) and gas chromatography (GC) analyses and quantified according to the methods described by Vanhercke et al. (2014).

### Total β-Glucan

Rice β-glucan was measured with reference to the methods in AOAC Method 995.16 (AOAC International 2016).

Briefly, 20 mg of rice flour were subjected to sequential enzymatic digestions of Lichenase and β-glucosidase, followed by quantification of the released glucose with standard glucose oxidase/peroxidase (GOPOD) system. Firstly, 200 µl 50% ethanol was added into 20 mg of rice flour, then 1 ml sodium phosphate buffer (20 mM, pH6.5) was added and incubated at 100 °C boiling water for 3 min. Secondly, the reaction was briefly cooled and diluted. Thirdly, 10 µl Lichenase (1U/µl) was added and incubated at 40 °C for 1 h. The sample was then diluted by adding 3.8 ml distilled water followed by centrifugation. Fourthly, the β-glucosidase reaction was conducted by adding 10 µl of β-glucosidase (2U/ml) into 10 µl sample supernatant collected in last step and incubated at 40 °C for 15 min.

Quantification of the glucose through GOPOD was conducted after the enzymatic reactions. Firstly, in 20 µl sample, 150 µl of glucose oxidase/peroxidase (GOPOD) reagent was added and incubated at 40 °C for 20 min. Secondly, the absorbance was measured at 510 nm for each sample (E_A_) and reagent blank (E_BLANK_). The β-glucan content was measured using the following formula:

$$\begin{aligned}\upbeta - {\text{glucan}}\,(\% {\text{Weight}}/{\text{Total}}\,{\text{weight}}) & = \Delta {\text{E}} \times {\text{F }} \times { 6}00 \times {1}/{1}000 \times {1}00/{\text{W}}_{{\text{d}}} \times {162}/{18}0 \\ & = \Delta {\text{E}}/\Delta {\text{G}}/{\text{W}}_{{\text{d}}} \times {135} \\ \end{aligned}$$

in which

∆E = the difference between E_A_ and E_BLANK_, F i = the absorbance of 2.5 µg of glucose, and W_d_ = weight of sample analysed (in mg) times 0.86 (W_d_ × 0.86).

## Results

### The Time Course of Aleurone Development in *ta2-1*

**Fig. 1:**
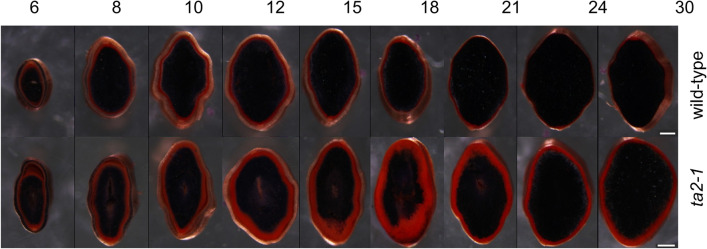
Sudan red and Lugol’s iodine staining showing the overall lipid and starch organization of rice caryopsis during seed development. Upper panel indicates the wild-type while lower panel indicates *ta2-1* at 6, 8, 10, 12, 15, 18, 21, 24, and 30DAA. Scale bar = 0.5 mm

To compare the aleurone development in *ta2-1* and wild type, caryopses of 6, 8, 10, 12, 15, 18, 21, 24, and 30DAA (days after anthesis) were examined. These time points represent important developmental phases such as aleurone cell fate differentiation, aleurone nutrient accumulation, and aleurone maturation. These caryopses were sectioned and stained with Sudan red plus Lugol’s iodine. The aleurone cells in *ta2-1* become prominent between 6 to 8DAA, being positively stained red by Sudan red (Fig. 1) indicating the dominance of lipid and almost complete absence of starch. Wild type had a thick aleurone resembling *ta2-1* up to 10DAA. From 12DAA onwards, *ta2-1* showed a thicker Sudan red staining area, although a slight decline of red staining area at 24 and 30DAA. Although there is no indication that starchy endosperm cells can become aleurone, it seems possible that some cell layers that initially show the compositional attributes of aleurone can become starchy endosperm in the later stages of development. In maize, *dek1* mutation triggered the transdifferentiation of the aleurone to starchy endosperm cells in the late stage of development (Becraft and Yi 2011). Furthermore, this switch is more pronounced in wild type caryopses than in *ta2-1*, leaving *ta2-1* with a thicker aleurone at maturity.

### General Cell Structure of *ta2-1*

To assess the cellular and subcellular identities of aleurone cells in rice *ta2-1*, light microscopy coupled with two different staining methods were adopted.

**Fig. 2:**
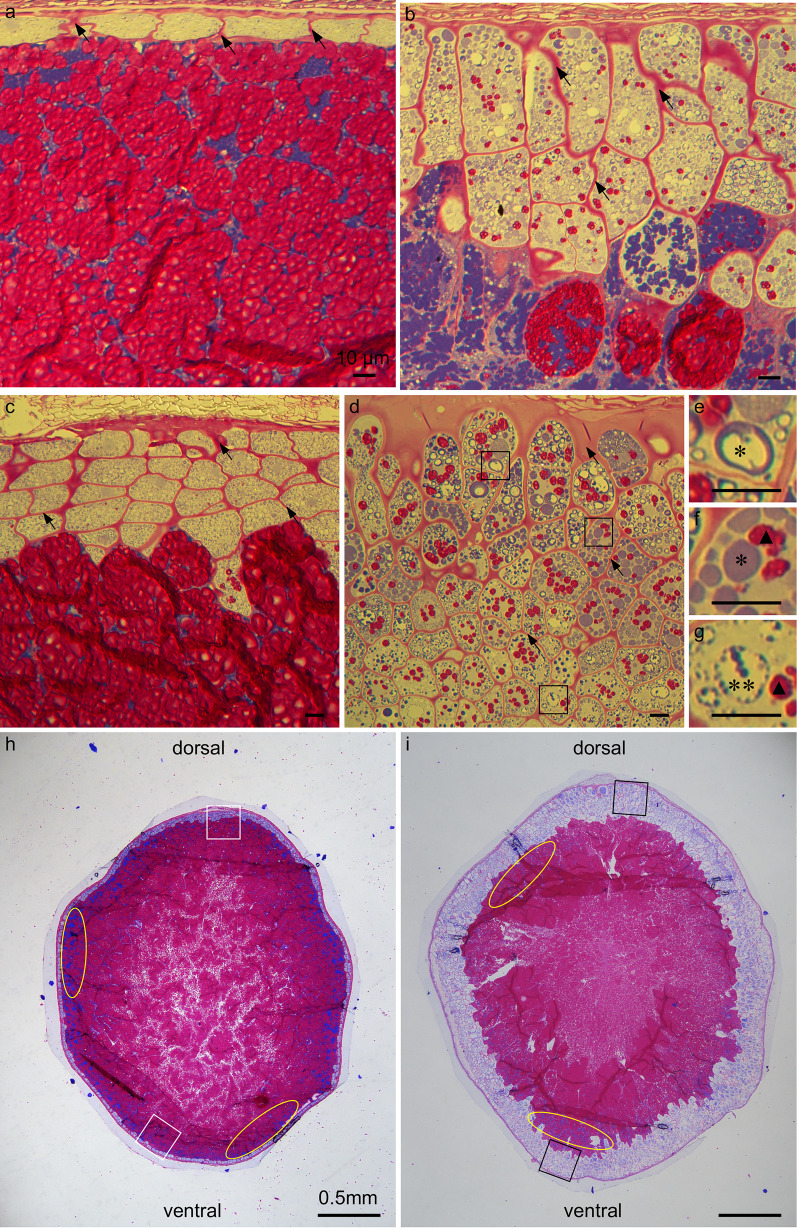
PAS and CBB staining showing the subcellular cell content of the transverse section of the rice caryopsis. PAS binds to and stains carbohydrate magenta while CBB binds to and stains protein blue. Different types of cell present across the putative multiple-aleurone layers in *ta2-1.* **a** and **c** the overview of the wild-type aleurone layers on the ventral side (**a**) and dorsal side (**c**) of rice caryopsis; **b** and **d** the overview of the *ta2-1* aleurone layers on the ventral side (**b**) and dorsal side (**d**) of rice caryopsis; **e**–**g** the aleurone cells in the *ta2-1* outer (**e**), middle (**f**), and inner (**g**) multiplied cell layers in three different regions of **d**. **h** and **i** semi-thin transverse section of the rice caryopsis showing the aleurone and starchy endosperm tissue layers in wild type (**h**) and *ta2-1* (**i**). In **a**–**d**, black arrows indicate the cell wall. In **d**, black square indicates the locations to capture the images used in **e**–**g**. In **e**–**g**, a single asterisk indicates the protein body identified in *ta2-1* resembling wild-type structure. Double asterisks indicate the large protein body in *ta2-1*. Black closed triangle indicates the starch granules presented in the multiple aleurone layers of *ta2-1*. In **g**, **h**, white square indicates the estimated locations to capture the images used in **a** (ventral) and **b** (dorsal). Black square indicates the estimated locations to capture the images used in **b** (ventral) and **d** (dorsal). Yellow oval indicates the sub-aleurone layer. Scale bar for **a**–**g** = 10 µm. Scale bar for **h**, **i** = 0.5 mm

Firstly, PAS-CBB staining, multiple layers of putative aleurone cells appeared distinctly different from starchy endosperm cells at the periphery of the mature rice caryopsis in *ta2-1*. The multiple layers of putative *ta2-1* aleurone cells had thicker cell walls (stained magenta by PAS) than the inner starchy endosperm cells (Fig. 2b, black arrow), prominent protein bodies (stained blue by CBB) (Fig. 2e, asterisk), and much lower starch granule density (Fig. 2f, g, black closed triangle). The putative aleurone cells in *ta2-1* were more irregular in shape than wild type aleurone cells, and with more and larger protein bodies. Moreover, their cell distribution patterns were different. In wild type, there was one aleurone layer on the ventral side (Fig. 2a) and up to five aleurone layers on the dorsal side of the rice caryopsis (Fig. 2c). In *ta2-1*, the aleurone layer was about three layers thick on the ventral side (Fig. 2b) and more than eight layers (Fig. 2d) on the dorsal side. Regarding the tissue organization, throughout the whole caryopses, *ta2-1* had more blue-stained aleurone-like area and less magenta-stained starchy-endosperm area than wild type (Fig. 2h, i). Between aleurone and starchy endosperm, protein bodies-rich sub-aleurone cell layer is present (Wu et al. 2016). In both *ta2-1* and wild type, heavily blue-stained sub-aleurone cell layers were observed (Fig. 2h, i, yellow oval). In the sub-aleurone cell layer, a mixture of lightly blue-stained aleurone cell, heavily blue-stained sub-aleurone cell, and magenta-stained starchy endosperm cell could present together (Fig. 2b).

**Fig. 3:**
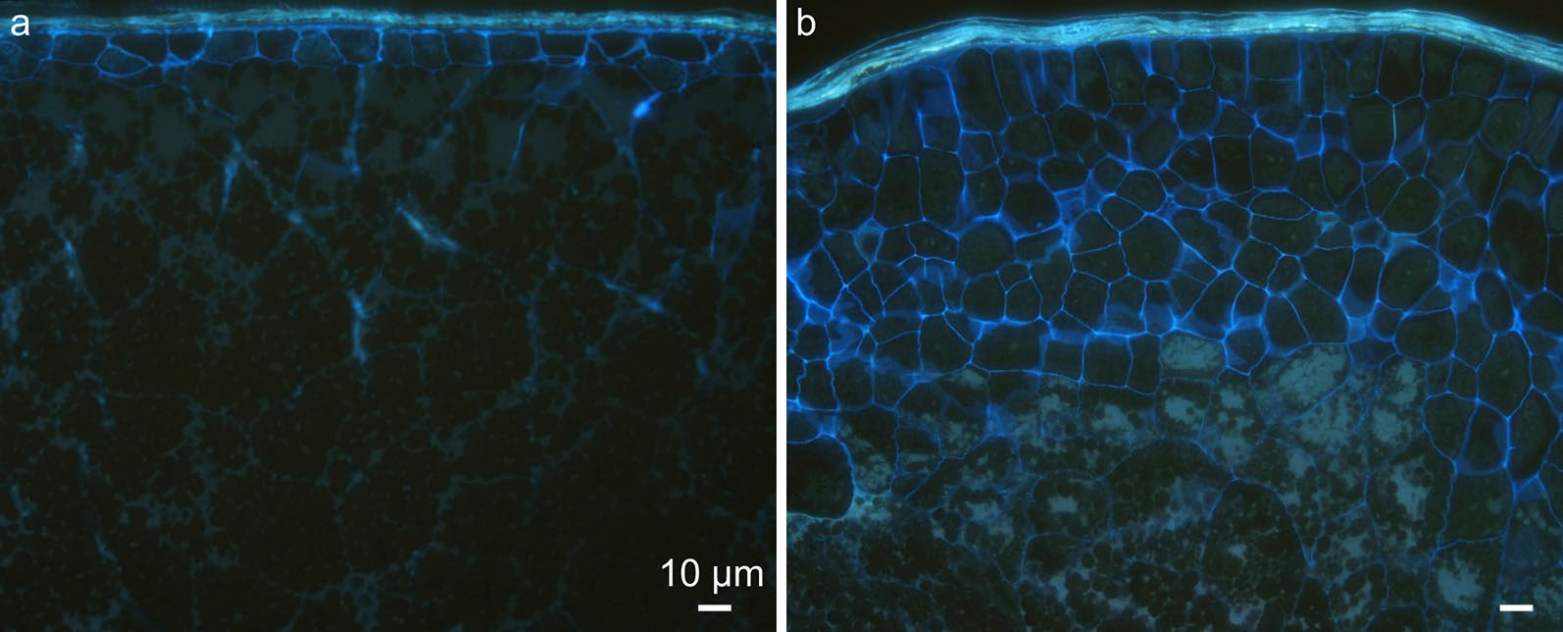
Calcofluor White staining with epifluorescence showing the cell wall structure and organization of the transverse section of the rice caryopsis. **a** the wild type; **b** *ta2-1*. Scale bar = 10 µm

Secondly, Calcofluor White staining was applied with epifluorescence microscopy to study the cell wall structure specifically. Calcofluor White binds to cellulose and other β-glucans in cell wall (Hughes and McCully 1975). In both wild type and *ta2-1*, the outermost compressed cells of the testa and the aleurone were actively stained while the innermost starchy endosperm cells were not (Fig. 3a, b). The putative extra aleurone layers of *ta2-1* resembled aleurone-like distinctive cell wall trait.

### The Accumulation of Aleurone Grains in *ta2-1*

**Fig. 4:**
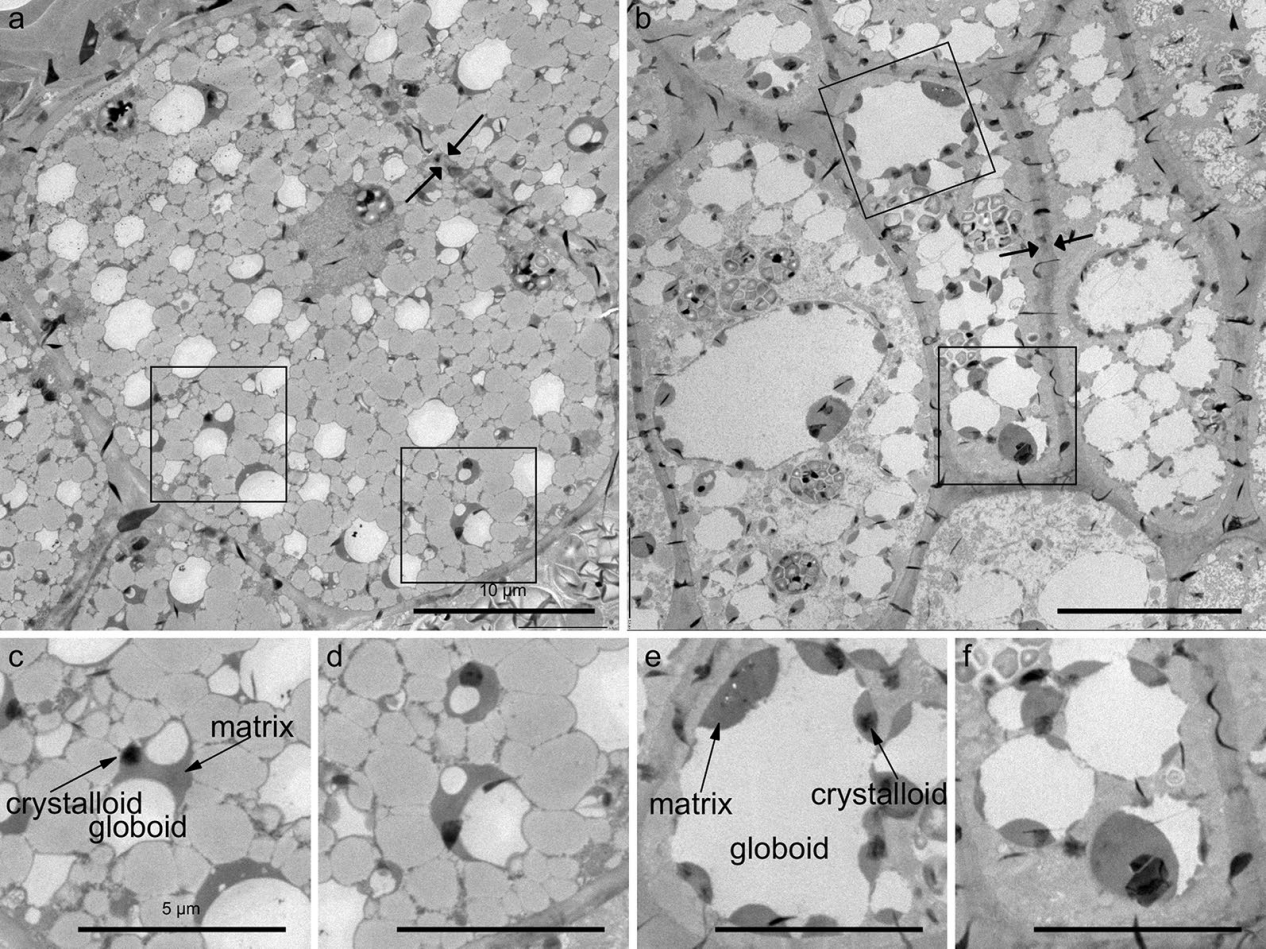
Transmission electron microscopy (TEM) showing the aleurone cell structure of the transverse section of the rice caryopsis. **a** wild-type; **b** *ta2-1*. The thick cell wall is shown between the arrows and aleurone grains (AG) are selected with black square. **c**–**f** magnified images selected as AG; **c** and **d** wild-type AG; **e** and **f** *ta2-1* AG. Scale bar for **a** and **b** = 10 µm. Scale bar for **c**–**f** = 5 µm

Transmission electron microscopy was used to examine the subcellular details of the putative aleurone cells in *ta2-1*. In both wild-type and *ta2-1* aleurone cells, thick cell walls were evident (Fig. 4a, b between two arrows). Also, protein bodies (sometimes referred to as aleurone grains (AG)) were abundant in both genotypes. As in the light microscopy study, AG in *ta2-1* were generally larger than the wild type with similar subcellular content. The *ta2-1* AG had other more subtle differences compared to wild type AG. Surrounded by tonoplast membrane, AG accumulate protein presumably as a source of amino acids for germination; they consist of three morphologically distinct features called the crystalloid, matrix, and globoid. These three features were present with similar electron density in the aleurone grains of both the wild type aleurone and the expanded *ta2-1* cells (Fig. 4c, e, arrows), however, the *ta2-1* AG were more variable in size with many being larger. When AG structures were selected and compared (Additional file 1: Fig. S1), 423.41µm^2^ and 251.83µm^2^ of AG area was observed in *ta2-1* and wild type respectively (Additional file 2: Table S1), suggesting that *ta2-1* had more AG area.

Together, the results from light microscopy and electron microscopy supported the conclusion that the putative extra aleurone cells in *ta2-1* retained the aleurone cell features of thick cell wall, low content of starch granules and presence of protein-rich AG. Distinctive AG features of crystalloid, matrix, and globoid were conserved in *ta2-1*. However, the *ta2-1* aleurone cells had a greater variability in cell size and shape of AG.

To measure the spatial distribution of aleurone cells, light microscopy coupled with PAS was adopted. From the outer to inner layers of the *ta2-1* aleurone, there was a change in structure and subcellular content of the AG. In wild type, only one type of AG was observed. The wild-type AG had a small internal cavity and abundant protein matrix that was stained deep blue by CBB. Moreover, individual aleurone cells were smaller in size (Fig. 2a, c) to those in *ta2-1* (Fig. 2b, d) The subcellular content and structure of the wild-type AG was uniform and constant. However, in *ta2-1*, two types of AG could be distinguished, i.e. AG with wild type morphology (Fig. 2e, f, single asterisk) and larger aleurone grains (LAG) (Fig. 2g, double asterisks). AG and LAG had similar deep-blue protein matrix feature, however, LAG had a larger internal globoid cavity with sometimes more limited protein matrix than AG. Moreover, the distribution patterns of LAG changed from outer to inner layers of the *ta2-1* aleurone. In the outer layers of *ta2-1* aleurone, the dominant storage compartment is AG while in inner layers, the dominant storage vacuoles resemble the LAG structure.

### The Contents of Dietary Fibre, Phenolic Compounds, and Antioxidants

**Fig. 5:**
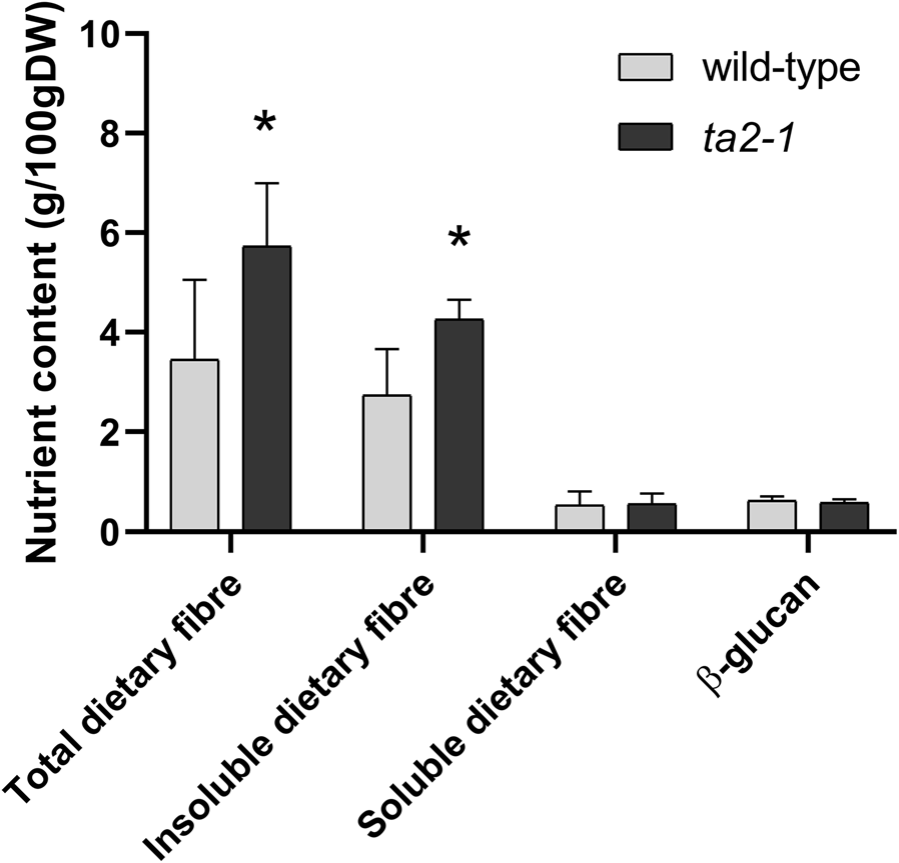
Total, insoluble, soluble dietary fibre, and β-glucan comparison (*P < 0.05; ANOVA, Tukey’s HSD test, total dietary fibre, n = 8; insoluble dietary fibre, n = 5; soluble dietary fibre, n = 5; β-glucan, n = 3). The exact p-value of all the statistical analyses in this study can be found in the file of Fig. 5-12_RawData.xlsx in the supplementary information

The aleurone cell wall in brown rice is an abundant source of dietary fibres. We previously showed that total dietary fibre content was increased in *ta2-1* (Liu et al. 2018). In this study, we compared the insoluble- and soluble- dietary fibre in the total fibre content of *ta2*-1. Similar to our previous findings, rice mutant *ta2-1* showed 66% increase in total dietary fibre. This was largely due to a 55% increase in insoluble-dietary fibre, while no significant change (*P* = 0.90 > 0.05) was observed in the content of soluble-dietary fibre (Fig. 5). Since it is known that the fibre associated with the aleurone cell wall is insoluble in nature (Collins et al. 2010), the observed increase in total and insoluble fibre in *ta2-1* is consistent with the thickened aleurone being the source of the extra fibre.

**Fig. 6:**
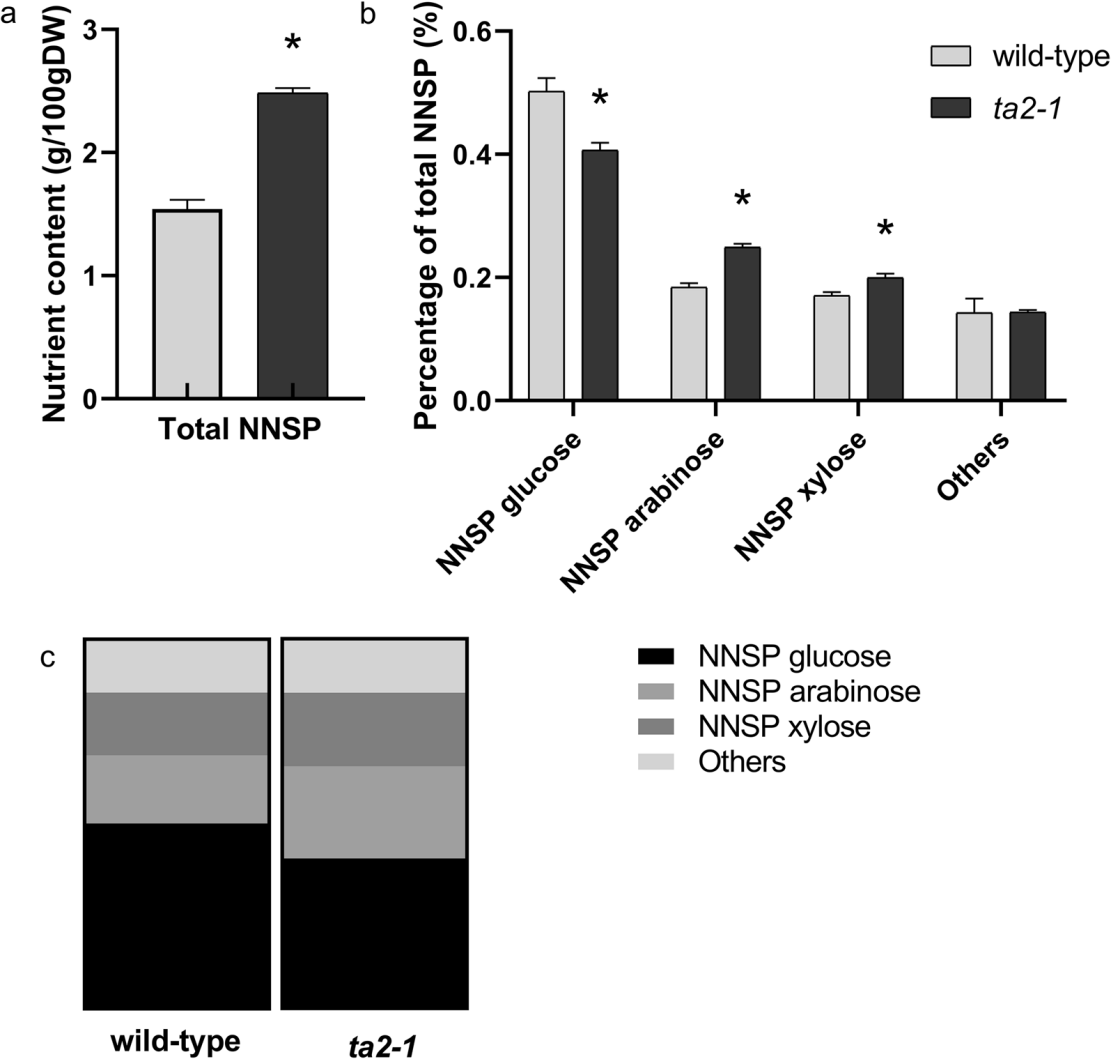
Comparison of total NNSP and NNSP composition. Comparison of **a** total NNSP, **b** NNSP composition of glucose, arabinose, and xylose (*P < 0.05; ANOVA, Tukey’s HSD test), and **c** NNSP composition in parts of whole format (total NNSP, NNSP glucose, arabinose, and xylose, n = 5)

Dietary fibre is composed NNSP, nondigestible oligosaccharide, resistant starch, lignin, and uronic acid containing polysaccharides (Theander et al. 1995). Among these components, NNSP is the major contributor of the total dietary fibre content in rice caryopsis (Collins et al. 2010). The total NNSP content in the wholegrain flour of *ta2-1* was 61% higher than that in the wild-type (Fig. 6a). Moreover, being consistent with the earlier studies in different wholegrain (brown) rice varieties (Collins et al. 2010; Demirbas 2005), the β-glucan content in the wild type and *ta2-1* was very low at 0.63 g and 0.59 g per 100 g respectively (Fig. 5). Aleurone is rich in arabinoxylan and low in cellulose and β-glucans, but starchy endosperm has an even distribution of these three fibre types (Fincher and Stone 2004). The arabinose content increased from 18% in the wild type to 24% in *ta2-1*, and the xylose content from 17 to 20%; while glucose content dropped from 50% in the wild type to 40% in *ta2-1* (Fig. 6b), leading to changed NNSP composition profile in *ta2-1* (Fig. 6c). The basic monosaccharide units of arabinoxylan are arabinose and xylose, and the basic monosaccharide unit of cellulose and β-glucans is D-glucose. The increase in arabinose and xylose content at the expense of D-glucose in NNSP suggested that the expanded aleurone layers in *ta2-1* maintained the aleurone cell identity of arabinoxylan-rich cell wall and accounted for the composition change in total NNSP.

Antioxidants of rice include a wide spectrum of biomolecules such as phenolic compounds, uronic acids, tocopherols, carotenoids, ascorbic acid, and gamma-oryzanol which stabilize multiple oxidant sources and free radicals by their electron-transferring or hydrogen-transferring ability (Prior et al. 2005). Some antioxidants can be esterified and bound to the aleurone cell wall. Our previous studies focused on the electron-transferring ability of wholegrain rice flour through oxygen radical absorbance capacity (ORAC) and the hydrogen-transferring ability through FCR (Liu et al. 2018). Here we tested their association with the aleurone cell wall, by extracting antioxidant fractions with different solubility and measuring their hydrogen-transferring abilities.

**Fig. 7:**
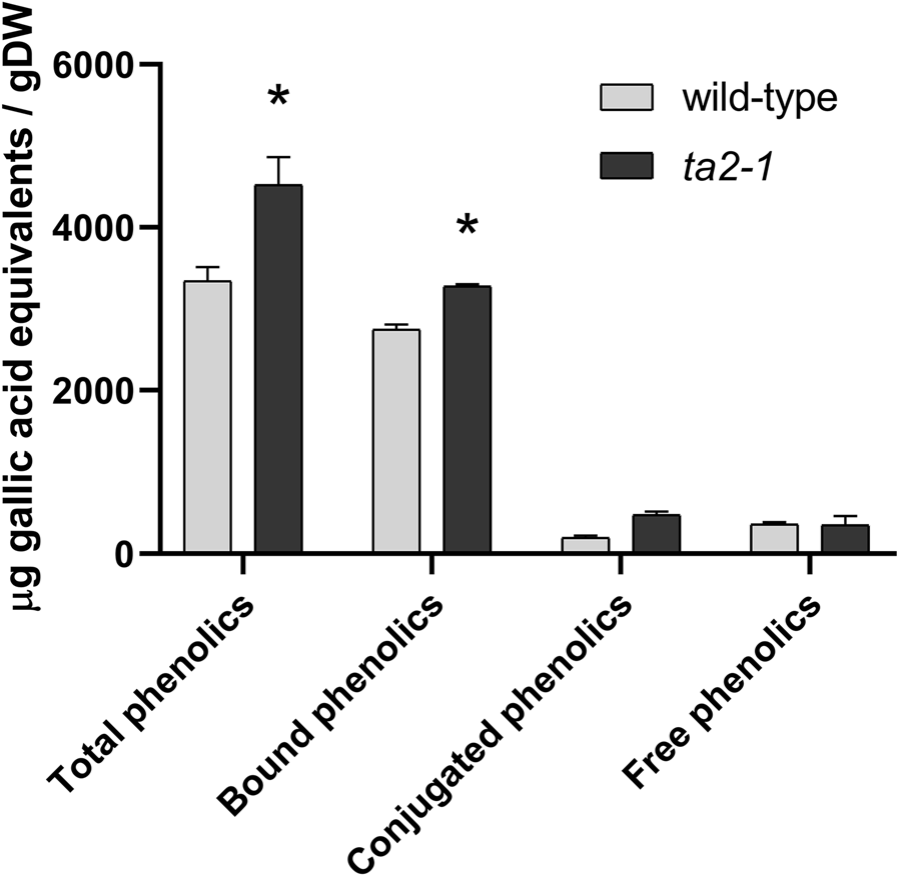
Phenolics composition of total, bound, conjugated, and free phenolics comparison (*P < 0.05; ANOVA, Tukey’s HSD test; total, bound, conjugated, and free phenolics, n = 3, except wild-type conjugated phenolics, n = 2)

Total-phenolic compounds in plants may exist in soluble free, soluble conjugated (esterified), and insoluble bound forms (Robbins 2003). The antioxidants were first extracted into the four fractions of total-, bound-, conjugated-, and free- phenolic compounds from the wholegrain rice flour samples. Their anti-oxidation abilities were then measured by the FCR assay. In both wild-type and *ta2-1*, about 80% of total phenolic compounds in rice caryopsis were insoluble bound-phenolic compounds, followed by 10% soluble free- and 10% soluble conjugated-phenolic compounds. Compared with the wild-type, *ta2-1* showed 35% increase in total-, 19% increase in insoluble bound-, no change in soluble free-, and 142% increase in soluble conjugated-phenolic compounds (Fig. 7). This pattern of increases is consistent with the increased aleurone thickness being responsible for the increased antioxidant capacity.

**Fig. 8:**
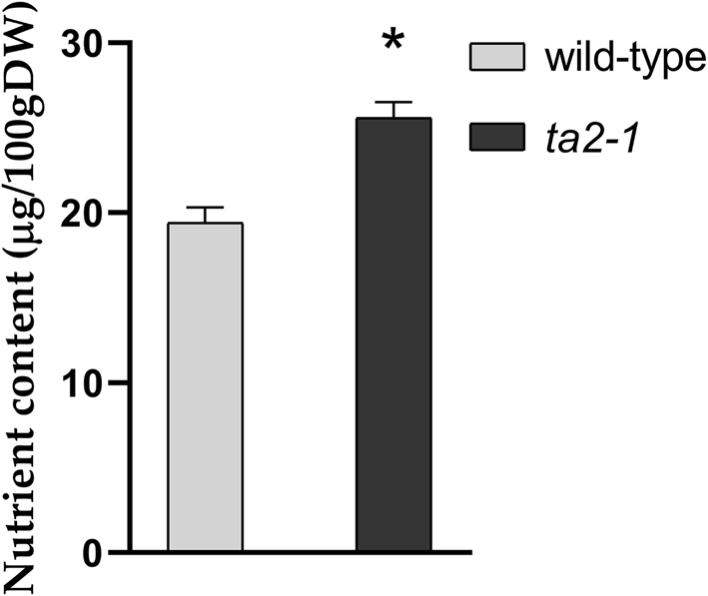
Folate (Vitamin B9) content comparison (*P < 0.05; ANOVA, Tukey’s HSD test; n = 3)

Folate (Vitamin B9) has potent antioxidant activity (Atteia et al. 2009). In rice, the aleurone-enriched bran fraction is rich in folate (Houston and Kohler 1970). In this study, compared with the wild-type, *ta2-1* had 32% increase in folate content (Fig. 8).

### The Contents of Minerals and Phytate

**Fig. 9:**
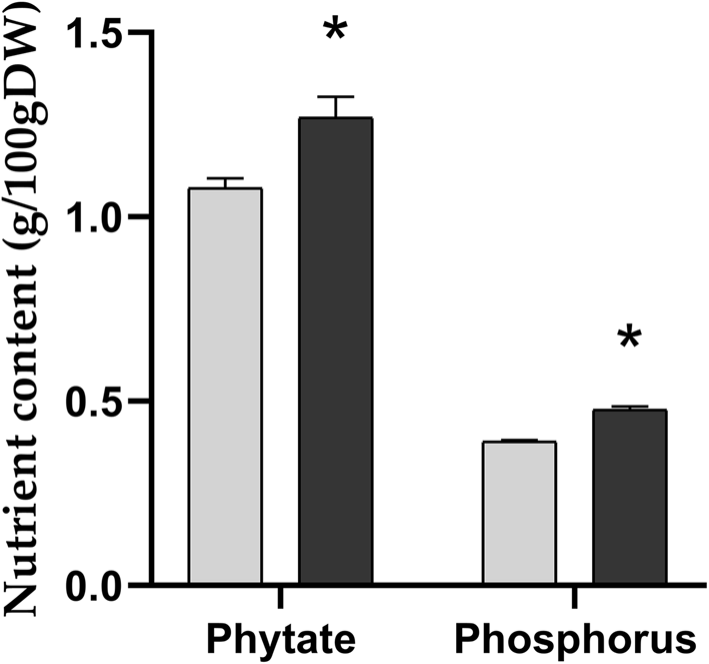
Total phytate comparison by Harland and Oberleas method and total phosphorus comparison by ICP-OES (*P < 0.05; ANOVA, Tukey’s HSD test; total phytate, n = 3; total phosphorus, n = 4)

**Fig. 10:**
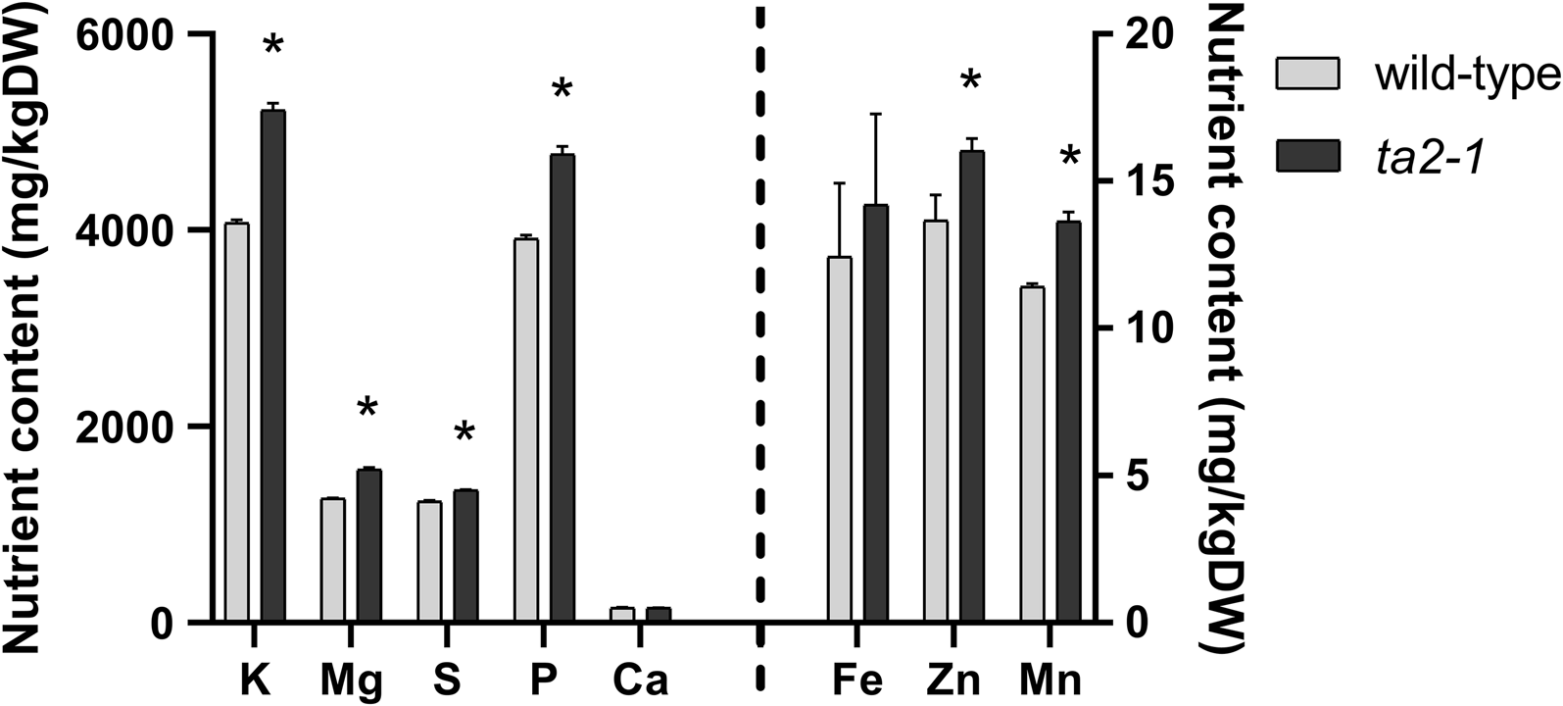
Minerals comparison. Potassium (K), Magnesium (Mg), Sulphur (S), Phosphate (P), and Calcium (Ca) content in the left panel follows the primary y-axis ranging from 0 to 6000 mg/100gDW (dry weight); Iron (Fe), Zinc (Zn), and Manganese (Mn) content in the right panel follows the secondary y-axis ranging from 0 to 20 mg/100gDW (*P < 0.05; ANOVA, Tukey’s HSD test; K, Mg, S, P, Ca, Fe, Zn, and Mn, n = 4)

Analyses of phytate and phosphorus content revealed increases of 18% and 22% respectively in *ta2-1* compared to wild type (Fig. 9). Assuming the molecular mass of phytate is 660.04gmol^−1^ and that of phosphorus is 30.97gmol^−1^ and each phytate molecular consists of six phosphorus molecules, 75.10% and 72.47% of total phosphorus are bound to phytate in wild type and *ta2-1* respectively (Additional file 3: Table S2). These measurements resemble the finding of others in rice caryopsis that 73.7% of total phosphorus content is bound to phytate (Ravindran et al. 1994). The similarity of percentage increase between phytate (18%) and phosphorus (22%) and the similar proportion of phosphorus bound to phytate suggested the increase of phosphorus in *ta2-1* is fully explained by the increase in phytate. Moreover, in addition to the increases in iron, zinc, and magnesium as observed by Liu et al. (2018), increases in manganese, potassium, and sulphur but not calcium content was observed (Fig. 10).

### The Compositions and Contents of Lipid

**Fig. 11:**
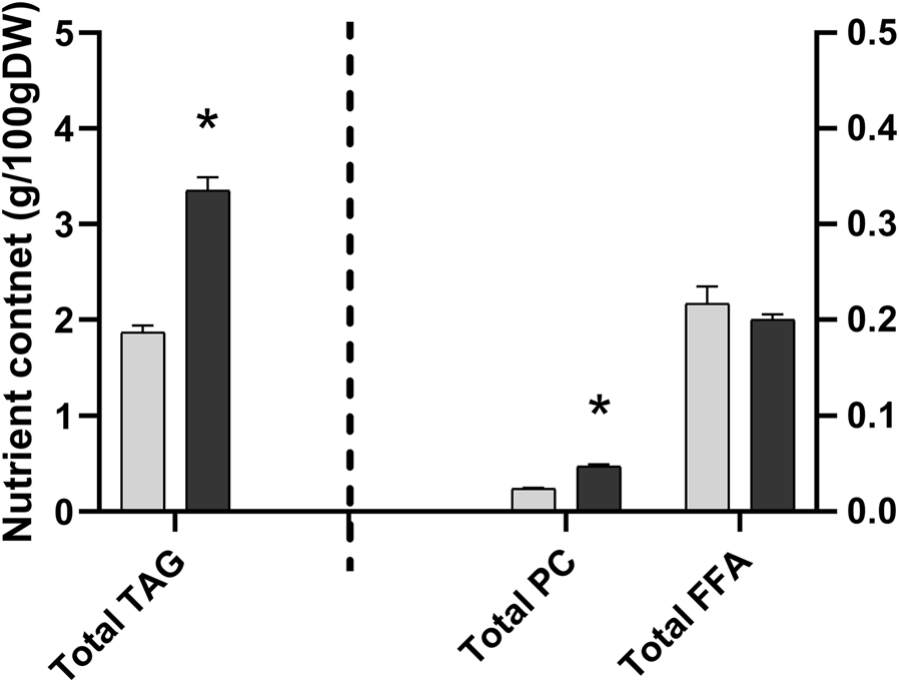
Lipid composition comparison. TAG content in the left panel follows the primary y-axis ranging from 0 to 5 g/100gDW; PC and FFA content in the right panel follows the secondary y-axis ranging from 0 to 0.5 g/100gDW (*P < 0.05; ANOVA, Tukey’s HSD test; TAG, PC, and FFA, n = 3)

**Fig. 12:**
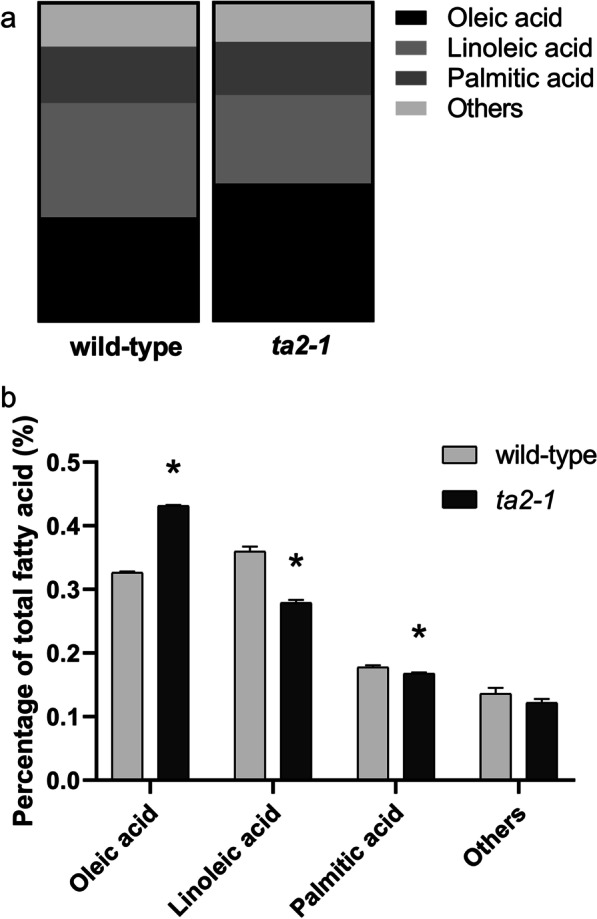
Fatty acid and fatty acid composition comparison. **a** Fatty acid composition comparison in parts of whole format, **b** Fatty acid composition comparison of oleic, linoleic, and palmitic acid (*P < 0.05; ANOVA, Tukey’s HSD test; oleic, linoleic, and palmitic acid, n = 4)

It has been shown before that *ta2-1* mutant had higher total lipid content (Liu et al. 2018). The total oil content and composition in rice aleurone (rice bran) is different from starchy endosperm (Choudhury and Juliano 1980a, 1980b). These authors showed that the neutral lipids (largely TAG) were concentrated in the bran (embryo and aleurone), while the phospholipids were equally distributed in the bran and starchy endosperm fractions. In our studies, lipid components were separated using thin-layer chromatography and fatty acid content quantified by gas chromatography. In both *ta2-1* and wild type, TAG was the dominant type of lipid, followed by FFA, and PC. In comparison with wild type, *ta2-1* had a 79% increase in TAG (from 1.89 to 3.32%), 97% increase in PC (from 0.02390 to 0.04715%), and a 7% decrease in FFA (from 0.2171 to 0.2004%; not statistically significant) in wholegrain rice flour samples (Fig. 11). The large increase in TAG is expected if the thick aleurone is responsible for the increase in total lipid. There were changes in fatty acid profile in *ta2-1* (Fig. 12a), including 32% increase in oleic acid content (from 32 to 42% of the total fatty acid), 22% decrease in linoleic acid content (from 35 to 28%), and 5% decrease in palmitic acid content (from 17 to 16%) (Fig. 12b).

## Discussion

### General Aleurone Identity of the *ta2-1* Thick Aleurone

The aleurone cells in *ta2-1* maintained the aleurone-like features of thick cell wall, with few starch grains, an abundance of AG and lipid bodies, and as a consequence increases in dietary fibre, phenolic compounds, lipid composition, and changes to fatty acid profile matching what would be expected if the increases come from normal aleurone cells.

Cytohistology with Calcofluor white staining, cytohistology with PAS-CBB staining, and transmission electron microscopy all demonstrated a dramatic expansion of thick cell walls in the entire *ta2-1* aleurone. The AG in *ta2-1* aleurone cells were more abundant and had the same distinctive structures of crystalloid, matrix, and globoid as wild-type AG, however, they were more variable in size with many being larger than in wild-type aleurone due mainly to larger globoid.

The increase in dietary fibre content in brown rice flours of *ta2-1* was mainly due to insoluble-dietary fibre, the type of fibre that forms aleurone cell walls (Collins et al. 2010). The increase in arabinose and xylose content *in ta2-1* flour and decrease in D-glucose was also consistent with the predominance of arabinoxylan in aleurone cell walls (Fincher and Stone 2004).

The enhanced thick cell wall in *ta2-1* also changes the phenolic compound composition. Associated with the cell wall are the phenolic compounds such as ferulic acid and ρ-coumaric acid. They are mainly bound to NNSP (Goufo and Trindade 2014). In *ta2-1*, the increase in total phenolic compounds was mainly attributable to an increase in insoluble bound-phenolic compounds.

Sudan red staining confirmed the enrichment of lipid bodies in the thickened *ta2-1* aleurone. The increase in TAG and PC in *ta2-1* flour but slight decrease in FFA, was consistent with a shift to aleurone- (bran-) specific lipid composition. Lipids in rice can be classified into non-starch lipids and starch-associated lipids (Zhou et al. 2003). Rice aleurone (bran) contributes to about 40% (39–41%) of total non-starch lipids that mainly consist of TAG and PC, while starchy endosperm stores about 60% (48–71%) of total starch-associated lipids with FFA as one of the major components (Choudhury and Juliano 1980a, 1980b). In *ta2-1* flour, the 79% increase in TAG, 97% increase in PC, and the 7% decrease in FFA is consistent with the thick aleurone maintaining normal aleurone cell function and composition. On the other hand, as the starchy endosperm content is reduced in *ta2-1*, the content of starch lipid FFA decreases.

The enrichment of lipid bodies in *ta2-1* aleurone also modifies the fatty acid profile. The fatty acid composition profiles of aleurone (bran) and starchy endosperm are different. Rice aleurone is rich in oleic acid (36% of total fatty acid) and linoleic (37%) but low in palmitic acid (23%) content. In starchy endosperm, linoleic and palmitic acid content are higher (41% and 33% respectively) but the oleic acid content is lower (20%) (Choudhury and Juliano 1980a, 1980b). The 32% increase in oleic, 22% decrease in linoleic, and 5% decrease in palmitic acid proportions again indicates that the multilayer aleurone in *ta2-1* maintains the aleurone-specific fatty acid profile.

Aleurone cells are rich in mitochondria (Jones 1969). In this study, no direct measurement of mitochondria was conducted, however, the folate content may be indicative of mitochondria abundance. Folate is enriched in aleurone-enriched bran fraction (Houston and Kohler 1970). In plant, folate is synthesized in three subcellular compartments in which the final five steps are conducted in mitochondria (Gorelova et al. 2017). About 30 – 50% of folate in cells is stored in mitochondria to help maintain the mitochondrial DNA stability (Depeint et al. 2006). Therefore, the 32% increase in folate may indirectly indicate that the multilayer aleurone in *ta2-1* maintains the aleurone-like high mitochondrial content.

Together, the cell structural features, the increase in insoluble fibre and insoluble bound-phenolic compounds, shift in aleurone-specific NNSP profile, enrichment of TAG and PC but slightly reduction in FFA and increase in oleic fatty acid composition collectively support the hypothesis that the additional aleurone-like layers in *ta2-1* maintain the distinctive features and composition of aleurone cells. It’s the expansion of the aleurone layer that results in an increase of the nutrients that are associated with aleurone cell. However, these additional aleurone-like cells are irregular in cell size with larger globoid structure, which are different from wild-type aleurone cells.

The improvement of multiple nutritional factors in *ta2-1* can help prevention of NCDs. For example, dietary fibre can maintain gastrointestinal health by increasing fecal bulk, decreasing transit time, decreasing the gastrointestinal contact of foodborne carcinogenic compounds, binding to mutagens, and lowering colonic pH (Glitsø et al. 1998; Le Gall et al. 2009). The insoluble antioxidants bound to cell wall materials can provide an antioxidant environment contributing to protection of the colon tissues from cancer (Sengupta et al. 2001). Phytate has potential anti-neoplastic and antioxidant functions (Fox and Eberl 2002; Norhaizan et al. 2011). The multilayer aleurone rice with true aleurone composition can coordinately improve multiple nutritional factors to achieve diverse protective outcomes. In countries with rice-dominant diets, the consumption of wholegrain rice is very low (Cleveland et al. 2000; Harnack et al. 2003), but there is a growing trend from white rice to brown rice (with aleurone) consumption due to improved health consciousness and education (Selvam et al. 2017). By improving the nutrient composition of brown rice that is more readily accepted by the public, the multilayer aleurone rice *ta2-1* could therefore deliver substantial public health advantage without remarkably changing the dietary habits. Furthermore, light polishing of thick aleurone rice retains more aleurone than wild type rice and therefore also retains more of the nutrients.

### The Commensurate Increase in Minerals and Phytate Content

Wholegrain flour of *ta2-1* had an 18% increase in phytate content. Using ICP-OES, we confirmed and extended our previous findings (Liu et al. 2018), showing *ta2-1* also had 14 to 23% increased content of various minerals.

Aleurone is a concentrated source of many essential minerals. In synchrotron X-ray fluorescence microscopy imaging study of rice, iron, zinc, manganese, and copper were highly concentrated in the aleurone layer (Hansen et al. 2012). Most of the minerals in rice are associated with phytate (Hansen et al. 2012; Mills et al. 2005; Simic et al. 2009). Wholegrain rice and wheat have similar phosphorus content (337 mg vs 323 mg/100gDW) (USDA 2020), however, rice has a higher proportion of phytate phosphorus than wheat, accounting for 77% of the total phosphorus content (Ravindran et al. 1994). Phytate has a strong affinity for chelating minerals such as Zn, Fe, and Mg, which limits their absorption in the small intestine (Bohn et al. 2007; Raboy 2009). This raises concern that phytate may impair small intestinal mineral absorption and compromise mineral status.

Wholegrain wheat flour has higher phytate and iron content than processed white flour, and in an in vitro* Caco-2* cells test, wholegrain wheat flour led to a lower ferritin response than processed white flour, suggesting that the iron content in white flour is more biologically available for cellular absorption and assimilation to ferritin (Eagling et al. 2014). Stevenson et al. summarized the findings in wheat and suggested that the consumption of wholegrain wheat or wheat bran decreased the calcium and zinc bioavailability (Stevenson et al. 2012).

However, the *Caco-2* test may not reveal the full picture concerning bioavailability. Diets high in whole grains do not adversely affect mineral nutrition but have favorable health outcomes. Different recommendations of daily wholegrain intake have been proposed in the U.S., Canada, and Australia, and no unfavourable health outcome has been reported regarding the high consumption of whole grains (Health Canada 2011; HHS & USDA 2015; National Health and Medical Research Council 2013). Recently, a meta-analysis provide further evidence that higher wholegrain consumption is associated with reduced risk of digestive tract cancers (Zhang et al. 2020). In both short-term (4 weeks) and long-term (2 years) studies in young and older women, the diet supplemented with phytate-rich wheat bran had no significant effect on different osteoporosis markers (Chen et al. 2004; Zittermann et al. 2007). Likewise, zinc absorption in young children was not negatively impacted by added phytate (Miller et al. 2015). Moreover, there is no consensus on the effect of phytate on iron bioavailability (Stevenson et al. 2012).

The digestion of phytate can happen in the human large intestine to release the chelated minerals for absorption. In pig studies, nearly complete (more than 97%) phytate digestion was observed in faecal samples following a normal diet with low intrinsic feed phytase (Schlemmer et al. 2001). In these studies, the highest phytate degradation occurred in the large intestine rather than the stomach or small intestine. In human faecal studies, the dietary phytate degradation rate varied between 50 and 90% (Joung et al. 2007; McCance and Widdowson 1935; Walker et al. 1948). In a human trial with both young and elderly women, it was also reported that the diet with high phytate content could enhance phytate degradation (Joung et al. 2007). In the large intestine, the microbial phytases, foodborne phytases in plant food sources, and endogenous phytases can degrade cereal-grain phytate to release the minerals bound in the mineral-phytate inclusion for human absorption (Sandberg and Andlid 2002). Moreover, the fermentation of the NNSP and different dietary fibres in rice can potentially acidify the luminal environment of the large bowel (Koh et al. 2016). This can improve the mineral bioavailability. Therefore, the commensurate increments of minerals and phytate in *ta2-1* may not necessarily decrease the mineral availability; on the contrary, the increased phytate content in *ta2-1* may stimulate the microbial phytate degradation in large intestine, releasing bioavailable minerals and digested phytate for human absorption and gut microbiome development. However, more studies regarding the minerals’ bioavailability should be conducted to further test this hypothesis.

### Why are the Nutrient Increases Not Greater?

The increases in multiple nutrients in *ta2-1* flour are significant but not in proportion to the increased layers or volume of aleurone cells. We briefly explore here two hypotheses, which are not mutually exclusive, that might explain this “nutrient gap”.

#### Hypothesis 1:

The new larger aleurone may have a lower capacity to synthesize and/or store nutrients compared to wild-type aleurone.

While we have shown the expanded aleurone in *ta2-1* has the general features and composition of normal aleurone, there may be more subtle differences in biosynthetic or storage capacity. In rice, both wild-type and *ta2-1* aleurone cells have prominent AG. However, the AG in *ta2-1* aleurone cells are more variable in morphology and distribution pattern from outer to inner layers.

The rice *ta2-1* had two types of AG, morphologically normal AG and LAG. While both types of aleurone grains had prominent globoid, crystalloid, and matrix structures, LAGs in *ta2-1* had enlarged globoid cavities. The crystalloid is the storage compartment for the integral membrane proteins, whereas the matrix contains soluble protein (Jiang et al. 2002, 2000). The globoid is the mineral storage compartment consisting of mineral-phytate inclusion crystals. Studies from energy-dispersive X-ray analysis in rice and synchrotron soft X-ray microscopy in wheat suggested that minerals and phosphorus are co-localized with the globoid structures in the AG (Ogawa et al. 1979; Regvar et al. 2011). However, during the sample preparation for transmission electron microscopy, the mineral-phytate crystals in the globoid can be easily dissolved out, thus leaving an electron-transparent internal globoid cavity in most of the electron microscopic analyses (Jacobsen et al. 1971). Despite these differences, some of the LAG in *ta2-1* accumulate protein that is stained by CBB, suggesting they are still functioning as storage compartments.

PAS and CBB staining also suggested that the distribution patterns of AG change from outer to inner layers of the *ta2-1* aleurone, with AG resembling wild-type structure in the outer layers, but LAG with empty or limited protein matrix predominant in the inner layers. The increased size of *ta2-1* aleurone cells and subcellular aleurone grains may signal a potential decrease in protein and mineral storage capacity from the outer to inner layers *ta2-1* aleurone.

#### Hypothesis 2:

The aleurone development does not synchronize with the nutrient accumulation during grain development.

From 3-5DAA is the endosperm cellularization stage, during which cells at the periphery first take on distinguishable aleurone features. From 6-9DAA, the aleurone and starchy endosperm cells continue to divide and expand during the endosperm differentiation stage (Olsen et al. 2008; Wu et al. 2016). The proportion of aleurone tissue to starchy endosperm tissue is high in these early stages as compared with the mid and late grain developmental stages with limited cell division. This may explain why thick-aleurone was observed in both wild type and *ta2-1* up to 10DAA. Our histological analyses suggested that aleurone development in *ta2-1* occurs simultaneously with aleurone in the wild type throughout all phases of grain development, so it is likely biosynthetic activity and nutrient accumulation will be synchronized with the normal activities during grain development.

Of these hypotheses, the first appears to have relevance regarding nutrients synthesized in the aleurone, and requires further investigation.

## Future Perspectives

The embryo is high in vitamin B1, vitamin E, and lipid, (Juliano 1993), so that any changes in embryo composition would also affect the overall wholegrain nutritional profile. Future studies should separate and study the composition of aleurone-, starchy endosperm-, and embryo- enriched fractions of *ta2-1*. It may also be possible to use synchrotron analysis to explore compositional differences between the outer and inner layers of the *ta2-1* thick aleurone, especially in mineral accumulation.

Future studies should also focus on assessing the bioavailabilities of minerals in *ta2-1*. Various digestion models such as in vitro* Caco*-2 cell, in vivo animal feeding trial, and human intervention studies should be applied to measure their absorption efficiencies along the gastrointestinal tract.

## Supplementary Information

**Additional file 1: Fig. S1**. Aleurone grain area comparison of TEM images. a: original image of wild-type in Fig. 4a; b: original image of ta2-1 in Fig. 4b. c and d: aleurone grain area was selected in red in wild type (c) and ta2-1 (d). Scale bar = 10µm.

**Additional file 2: Table S1**. Aleurone grain area comparison of TEM images.

**Additional file 3: Table S2**. Phosphorus content bound to phytate in rice wholegrain samples.

## Data Availability

The data generated or analysed during this study are included in (1) Supplementary information file in this article and (2) FigShare repository [https://figshare.com/s/0ae0f17be3bf75f2af4e] before publication. Once the manuscript has been accepted, public repository of [https://doi.org/10.6084/m9.figshare.12818867] will be available.

## Abbreviations

AG: Aleurone grain
BC3: Backcrossed three times
CBB: Coomassie brilliant blue
DAA: Days after anthesis
DW: Dry weight
FCR: Folin–Ciocalteu reagent
FFA: Free fatty acid
HSD: Honestly significant difference
LAG: Larger aleurone grains
NCD: Noncommunicable disease
NNSP: Neutral non-starch polysaccharide
ORAC: Oxygen radical absorbance capacity
PAS: Periodic acid-Schiff
PC: Phosphatidylcholine
*ta2-1*: *Thick-aleurone 2-1*
TAG: Triacylglycerol
TLC: Thin layer chromatography
ZH11: Zhonghua 11
