# Supplementary material for: Rice with Multilayer Aleurone: A Larger Sink for Multiple Micronutrients
Source: Rice (N Y). 2021 Dec 13;14:102. doi: 10.1186/s12284-021-00543-3 (PMC8669085; doi:10.1186/s12284-021-00543-3)
Supplement: Supplementary file 1 — Additional file 1: Fig. S1. Aleurone grain area comparison of TEM images. a: original image of wild-type in Fig. 4a; b: original image of ta2-1 in Fig. 4b. c and d: aleurone grain area was selected in red in wild type (c) and ta2-1 (d). Scale bar = 10µm. [file 12284_2021_543_MOESM1_ESM.docx]

| 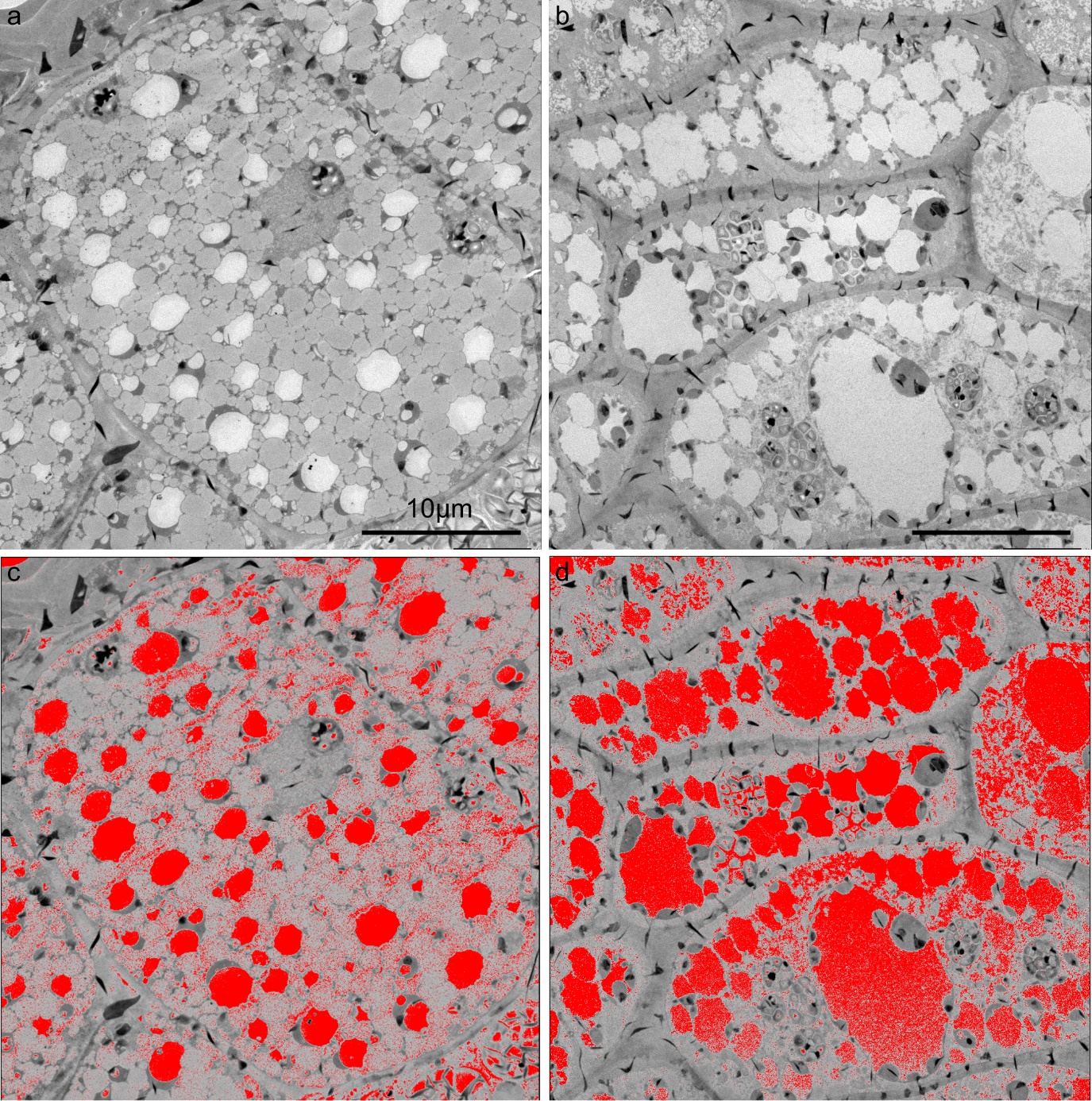  **Sup Fig. 1**  Figure title:  Aleurone grain area comparison of TEM images.  Figure legend:  **a**: original image of wild-type in Fig. 4a; **b**: original image of *ta2-1* in Fig. 4b. **c** and **d**: aleurone grain area was selected in red in wild type (c) and *ta2-1* (d). Scale bar = 10µm. |
| --- |
