# Supplementary material for: Rice with Multilayer Aleurone: A Larger Sink for Multiple Micronutrients
Source: Rice (N Y). 2021 Dec 13;14:102. doi: 10.1186/s12284-021-00543-3 (PMC8669085; doi:10.1186/s12284-021-00543-3)
Supplement: Supplementary file 2 — Additional file 2: Table S1. Aleurone grain area comparison of TEM images. [file 12284_2021_543_MOESM2_ESM.docx]

**Supplementary Table 1**

Aleurone grain area comparison of TEM images.

| **Sample** | **Aleurone grain area (µm^2^)^1^** | **Percentage Area (%)^2^** |
| --- | --- | --- |
| **ZH11** | 251.82 | 20.69 |
| ***ta2-1*** | 423.41 | 34.55 |

Supplementary table title:

Aleurone grain area comparison of TEM images.

Supplementary table legend:

Calculation of aleurone grain area as follows:

^1^ In the calibrated TEM images, the threshold was adjusted to between 196 and 255. This range covered most of the globoid area of the aleurone grain. The thresholded areas were selected and compared between *ta2-1* and wild type.

^2^ Percentage area = thresholded area / total area of the TEM image.
