# Supplementary material for: Rice with Multilayer Aleurone: A Larger Sink for Multiple Micronutrients
Source: Rice (N Y). 2021 Dec 13;14:102. doi: 10.1186/s12284-021-00543-3 (PMC8669085; doi:10.1186/s12284-021-00543-3)
Supplement: Supplementary file 3 — Additional file 3: Table S2. Phosphorus content bound to phytate in rice wholegrain samples. [file 12284_2021_543_MOESM3_ESM.docx]

**Supplementary Table 2**

Phosphorus content bound to phytate in rice wholegrain samples.

| **Sample** | **No. of mole of** | | | **P in phytate / Total P content** |
| --- | --- | --- | --- | --- |
|  | **Phosphorus^1^** | **Phytate^2^** | **P in Phytate^3^** |  |
| **ZH11** | 12.625X10^-3^ | 1.580 X10^-3^ | 9.481 X10^-3^ | 75.10% |
| ***ta2-1*** | 15.402X10^-3^ | 1.860 X10^-3^ | 11.16 X10^-3^ | 72.47% |

Supplementary table title:

Phosphorus content bound to phytate in rice wholegrain samples.

Supplementary table legend:

Calculation of phosphorus content is based on the equations as follows:

^1^ The molar mass of phosphorus is 30.97gmol^-1^, therefore, mole of phosphorus = mass of phosphorus / molar mass.

^2^ The molecular mass of phytate is 660.04 gmol^-1^, therefore, mole of phytate = mass of phytate / molecular mass.

^3^ As the molecular formula of phytate is C_6_H_18_O_24_P_6_, one mole of phytate contains six moles of phosphorus.
